# A novel and efficient fungal delignification strategy based on versatile peroxidase for lignocellulose bioconversion

**DOI:** 10.1186/s13068-017-0906-x

**Published:** 2017-09-13

**Authors:** Wen Kong, Xiao Fu, Lei Wang, Ahmad Alhujaily, Jingli Zhang, Fuying Ma, Xiaoyu Zhang, Hongbo Yu

**Affiliations:** 1 College of Life Science and Technology, Huazhong University of Science and Technology, Wuhan, 430074 People’s Republic of China; 2 College of Life Science and Technology, WuHan University of Technology, Wuhan, 430070 People’s Republic of China

**Keywords:** *Physisporinus vitreus*, Enzymatic recalcitrance removal, Versatile peroxidase, Corn stover, Biofuel, Lignin, 5-5′ linkage

## Abstract

**Background:**

The selective lignin-degrading white-rot fungi are regarded to be the best lignin degraders and have been widely used for reducing the saccharification recalcitrance of lignocellulose. However, the biological delignification and conversion of lignocellulose in biorefinery is still limited. It is necessary to develop novel and more efficient bio-delignification systems.

**Results:**

*Physisporinus vitreus* relies on a new versatile peroxidase (VP)-based delignification strategy to remove enzymatic recalcitrance of corn stover efficiently, so that saccharification of corn stover was significantly enhanced to 349.1 mg/g biomass (yield of glucose) and 91.5% (hydrolysis yield of cellulose) at 28 days, as high as levels reached by thermochemical treatment. Analysis of the lignin structure using pyrolysis–gas chromatography–mass spectrometry (Py–GC/MS) showed that the total abundance of lignin-derived compounds decreased by 54.0% and revealed a notable demethylation during lignin degradation by *P. vitreus*. Monomeric and dimeric lignin model compounds were used to confirm the ligninolytic capabilities of extracellular ligninases secreted by *P. vitreus*. The laccase (Lac) from *P. vitreus* could not oxidize nonphenolic lignin compounds and polymerized β-*O*-4 and 5-5′ dimers to precipitate which had a negative effect on the enzymatic hydrolysis of corn stover in vitro. However, the VP from *P. vitreus* could oxidize both phenolic and nonphenolic lignin model compounds as well as break the β-*O*-4 and 5-5′ dimers into monomeric compounds, which were measured by high-performance liquid chromatography–electrospray ionization–mass spectrometry (LC–ESI–MS). Moreover, we showed that addition of purified VP in vitro improved the enzymatic hydrolysis of corn stover by 14.1%.

**Conclusions:**

From the highly efficient system of enzymatic recalcitrance removal by new white-rot fungus, we identified a new delignification strategy based on VP which could oxidize both phenolic and nonphenolic lignin units and break different linkages in lignin. In addition, this is the first evidence that VP could break 5-5′ linkage efficiently in vitro. Moreover, VP improved the enzymatic hydrolysis of corn stover in vitro. The remarkable lignin-degradative potential makes VP attractive for biotechnological applications.

**Electronic supplementary material:**

The online version of this article (doi:10.1186/s13068-017-0906-x) contains supplementary material, which is available to authorized users.

## Background

The so-called second-generation biofuel technology aims to utilize the nonfood-based lignocellulosic biomass from agricultural by-products, perennial crops, forest residues, and pulp industries to avoid the food vs. fuel controversy [1]. Agricultural by-products, such as corn stover, are attractive feedstocks for the production of second-generation bioethanol because of their high abundance [2]. However, the presence of lignin in these lignocellulosic materials impedes the enzymatic digestibility of cellulose [3]. Thus, the degradation of lignin by thermochemical reaction is critical to remove enzymatic recalcitrance of lignocellulose and enhance the hydrolysis of cellulose [4, 5], but it is very expensive and produces a series of toxic compounds, such as black liquor which are harmful for the environment or human health, and furfural and 5-hydroxymethylfurfural which inhibit the downstream enzymatic hydrolysis and fermentation [6]. Therefore, biological delignification has been considered as an advantageous alternative, because it is low-cost, environmental friendly, and does not produce inhibitors to fermentation [7]. There are varied patterns of biomass components degradation by fungi. These patterns included predominant polysaccharide degradation (brown-rot), simultaneous degradation of all biomass components (white-rot), and selective degradation of lignin with cellulose preservation (selective white-rot) [8, 9]. Selective white-rot fungal pretreatment can improve enzymatic saccharification by degrading lignin. However, most biological delignification processes have very low hydrolysis yields [10, 11]; thus, novel and more efficient bio-delignification systems are necessary.

In nature, lignin degradation is a multi-enzymatic process involving an array of accessory enzymes (such as producing hydrogen peroxides) in addition to the four major lignilolytic enzymes. Different fungal species have been reported to exhibit different degradation capacities towards lignin, and their efficiency mainly depends on the ligninolytic enzymes produced by the white-rot fungi [12–14], mainly including laccase (Lac, EC 1.10.3.2), manganese peroxidase (MnP, EC 1.11.1.13), lignin peroxidase (LiP, EC1.11.1.14), and versatile peroxidase (VP, EC 1.11.1.16) [12]. The production and activity of these enzymes as well as their ability to degrade lignin vary significantly in different species of white-rot fungi. Differences in the catalytic properties of these enzymes strongly affect lignin degradation [14]. Lacs possess relatively low redox potentials (0.48–0.78 V) that restrict their action to the oxidation of the nonphenolic lignin components [15]. However, a typical lignin polymer is 10–15% phenolic in composition, rendering up to 80–90% of the nonphenolic polymer unreactive to Lac [16]. Although Lac mediator systems have extended the substrate range of Lac to include the oxidation of nonphenolic lignin model compounds, some studies demonstrated that the presence of 1-hydroxybenzotriazole (HBT), a common mediator, strongly favors the oxidation reaction pathways over the coupling reactions that lead to polymerization of lignin [17, 18]. Additionally, Lac does not cleave condensed structures, such as the 5-5′ model dimer, even in the presence of mediators [19]. Peroxidases exhibit a high redox potential (0.8–1.2 V), which is required for lignin modification and degradation of lignocellulosic biomass [15]. LiPs are strong oxidants that interact directly with nonphenolic lignin structures to cleave them but they do so inefficiently and apparently cannot penetrate the small pores in sound lignocellulose. MnPs produce small diffusible strong oxidants that can penetrate the substrate but they cleave the principal structures of lignin with low yields [13]. VPs are structurally LiP-like hybrid MnPs with a high redox potential, which combine the catalytic properties of LiPs and MnPs [20–22]. Thus, VPs possess extraordinarily wide substrate specificity and can oxidize both low and high redox potential compounds [23, 24]. The unique properties of VPs strongly suggest that they contribute to ligninolysis; however, the role of VPs in fungal delignification has not been reported before and their relative importance remains unclear. So far, VPs have not been used successfully to delignify intact lignocellulose in vitro.

*Physisporinus* sp., a white-rot basidiomycete, degrades lignin selectively and has a noteworthy biotechnological potential [25]. The white-rot fungus *Physisporinus vitreus* is currently tested for several biotechnological applications such as biopulping of softwood, permeability improvement of refractory wood species, and biopretreatment [26]. Its efficiency in these processes has been mainly attributed to the release of a battery of ligninolytic enzymes. Screening of the enzymatic activity during wood degradation shows that *P. vitreus* secreted high amounts of laccase, while the peroxidases (Lip, MnP, and VP) were not detected [27].

This study is the first to report a novel and efficient delignification strategy for enzymatic recalcitrance removal of corn straw based on VP from selective lignin-degrading fungus *P. vitreus*. The effect of fungal delignification on enzymatic hydrolysis was evaluated. In addition, we further characterized lignin degradation by VP using different monomeric and dimeric lignin model compounds. Finally, we studied the enhancement of the enzymatic hydrolysis of corn stover by VP in vitro.

## Results and discussion

### Enzymatic hydrolysis, component analysis, and enzyme production during pretreatment of corn stover with *P. vitreus*

**Fig. 1:**
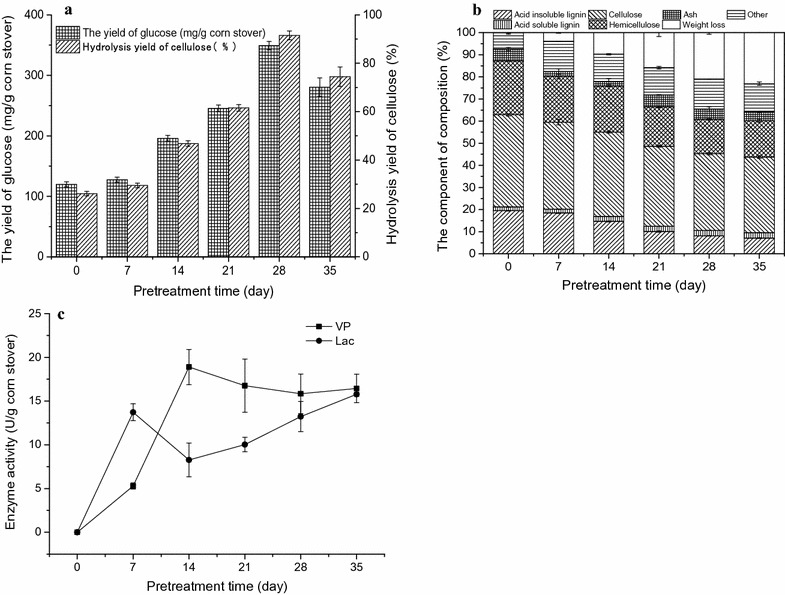
**a** The glucose yield and hydrolysis yield of cellulose after enzymatic hydrolysis of corn stover pretreated with *P. vitreus* for different times. **b** The component analysis of corn stover after *P. vitreus* pretreatment for different times. **c** Enzyme production during *P. vitreus* pretreatment of corn stover

As shown in Fig. 1a, corn stover without pretreatment were much more resistant to enzymatic hydrolysis and the yield of glucose only was 119.8 mg/g corn stover after 72-h hydrolysis. The conversion ratio of cellulose in raw corn stover was only 26.1%. Higher glucose yields and cellulose conversion ratio were achieved when the corn stover pretreated with *P. vitreus*. There was a rapid increase in the glucose yield and cellulose conversion ratio with increasing pretreatment time before 28-day pretreatment. At the same time, it can be easily seen that the weight loss of corn stover increased with an increasing pretreatment time. Thus, the glucose yield and cellulose conversion ratio after enzymatic hydrolysis were used to evaluate the effect of pretreatment by taking the weight loss into consideration. The maximum glucose yield and cellulose conversion ratio reached up to 349.1 mg/g corn stover and 91.5% after 28 days of pretreatment, increasing by 2.9 and 3.5 times, respectively, compared with untreated corn stover. The conversion of cellulose to glucose was higher than the fungal pretreatments reported previously [28, 29], and similar to that of biomass pretreated with thermochemical processes [5, 6, 30]. The hydrolysis yields of various substrates by different fungal strains reported previously are usually ranging from 30 to 83% [29]. The hydrolysis yields of straw pretreated with thermochemical processes are usually about 80–100% [5]. Compared to thermochemical pretreatment, biological delignification has been considered as an advantageous alternative, because it is low-cost, environmental friendly, and does not produce inhibitors to fermentation [7]. The efficiency of pretreatment depended on fungus species due to versatile delignification strategies of white-rot fungi, and the delignification abilities of different fungi species vary greatly [31]. To date, only several fungi strains can improve the saccharification efficiency significantly as high as levels reached by thermochemical treatment [28, 29]. Thus, development of novel and more efficient fungi strains like *P. vitreus* is necessary and meaningful.

Moreover, as shown in Fig. 1a, after increasing pretreatment time from 28 to 35 days, a decrease in hydrolysis yield is observed. This result can be explained by the fact that digestibility of cellulose is affected by both lignin removal and lignin modification [32]. Removal of lignin from lignocellulose increased substrate hydrophilicity and enlarged the volume of accessible pores. This allowed more cellulase to infiltrate into the lignocellulosic matrix to access cellulose [33, 34], thus improving saccharification of corn stover from 7 to 28 days of biological pretreatment. However, changes on the lignin surface take place during the pretreatment process that might increase the nonproductive absorption of cellulase could negatively influenced hydrolysis yields from 28 to 35 days of biological pretreatment [35, 36]. This effect will be further studied in detail in a future work.

As shown in Fig. 1b, fungal pretreatment preserved most of the cellulose and removed 63.6 and 32.6% of the lignin and hemicellulose, respectively. A major drawback of biological pretreatment processes is the potential loss in sugar content. *P. vitreus* selectively degrade lignin and consume only little sugar while preserving most of the cellulose content. Although some sugar was consumed by *P. vitreus*, the final yield of glucose was improved compared to the untreated corn stover. In lignocellulosic biomass, cellulose and hemicellulose are densely packed by lignin layers, which protect them against enzymatic hydrolysis [37]. The improvement of glucose production might be mainly attributed to the oxidation of lignin by *P. vitreus* [3, 38]. On one hand, fungal pretreatment can reduce saccharification recalcitrance of lignocellulosic biomass by lignin degradation [39]. On the other hand, biological pretreatment has also been shown to promote lignin modifications, such as changes in lignin hydrophobicity, which then decreased the unproductive adsorption of cellulase onto lignin [32]. In addition to the lignin degradation, there was a loss in hemicellulose content. Hemicellulose serves as a connection between lignin and cellulose fibrils and its degradation may contribute to reducing the natural recalcitrance of lignocellulosic substrates [40]. The decrease in hemicellulose content is likely a consequence of lignin degradation, which enhances the accessibility of hemicellulose for enzyme hydrolysis by xylanase [41]. Indeed, we detected xylanase activity (5 U/g corn stover) during the pretreatment with *P. vitreus*. Thus, the degradation of lignin promotes the degradation of hemicellulose, which also contributes to saccharification. The nonglucose sugar from hemicellulose might was a potential carbon and energy source for fungal growth. In general, fungal lignin oxidation plays an important role in improving saccharification yields of lignocellulosic substrates.

The ability of white-rot fungi to degrade lignin is mainly attributed to the release of a battery of ligninolytic enzymes [12, 13]. In order to analyze the high efficiency of this strain in enzymatic recalcitrance removal and degradation of lignin, we studied its extracellular ligninases (Fig. 1c). The extracellular extracts of pretreatment cultures at different periods were assayed to determine the different ligninolytic enzymes. Two main ligninolytic enzymes were detected during pretreatment of corn stover with *P. vitreus*. One was identified as Lac; the other was a special MnP, which could oxidize Mn^2+^ but was also able to oxidize veratryl alcohol (VA) and reactive black 5 (RB5) in the absence of Mn^2+^. This MnP was identified as a VP since it shares typical features of both MnP and LiP. For example, both VP and LiP oxidize VA, but only VP oxidizes Mn^2+^, RB5, and other dyes. To date, VPs had only been found in *Pleurotus* sp. [42, 43] and *Bjerkandera* sp. [44, 45]. This is the first report of a novel VP from white-rot fungus *Physisporinus* sp. As shown in Fig. 1d, VP displayed high activity from the 2nd week on and reached a maximum activity of 18.9 IU/g corn stover at 14 days of pretreatment. In contrast, Lac reached its maximum activity of 15.8 IU/g corn stover at later period, after 35 days of pretreatment. Interestingly, major lignin degradation occurred by day 21 of pretreatment (Fig. 1b), which implies that VP might play a key role in the degradation of lignin from *P. vitreus*.

### Py–GC–MS analysis of corn stover

To obtain a more detailed insight into the chemical modifications of the lignin structure after fungal pretreatment, the untreated and 28-day treated corn stovers were analyzed by Py–GC/MS. Additional file 1 shows the pyrograms of the untreated and 28-day treated samples, which were dominated by peaks of phenolic compounds derived from the lignin moiety. The identities and relative abundances of the lignin-derived compounds released are listed in Table 1. In both samples, syringyl- (S-) and guaiacyl (G-)-type phenols were released, with a predominance of the latter and similar distribution patterns, together with minor amounts of *p*-hydroxyphenyl (H-)-type phenols, which is in agreement with previous reports [46, 47]. After fungal pretreatment, the total abundance of lignin-derived compounds decreased by 54.0%, which means that fungal treatment led to a considerable degradation of lignin. This is consistent with the component analysis of lignocellulosic materials after fungal pretreatment. In addition, the relative contents of most S-, G-, and H-type lignin derivatives decreased and even disappeared. For example, guaiacol (peak 4) derived from G-type lignin decreased by 49.5% and syringol (peak 14) derived from S-type lignin decreased by 31.2% after fungal treatment, while 4-methylguaiacol (peak 7), 4-ethylguaiacol (peak 9) derived from G-type lignin and 3,5-dimethoxyacetophenone (peak 23), 3,5-dimethoxy-4-hydroxycinnamaldehyde (peak 30) derived from S-type lignin disappeared. After fungal delignification, the total peak area of H-, G- and S-type lignin derivatives was reduced by 44.0, 52.5, and 60.1% respectively, indicating that *P. vitreus* preferentially degrades S-type units, followed by G-type units, and is least efficient at degrading H-type units. Thus, the presence of more methoxy groups in the lignin correlated with a higher degradation rate by *P. vitreus*, which agrees with the finding of our previous study [48]. Compared with the raw sample, the treated sample contained less S-type lignin derivatives, which means that fungal delignification could have led to a substantial demethoxylation of lignin, and part of the G and H units in the treated sample might be derived from the fungal demethoxylation of S units [49].

**Table 1:** Relative peak areas (%) of lignin-derived compounds identified by analytical pyrolysis

Label	Compound	Origin	Peak area (%)	
			Corn stover	Fungal-treated corn stover
1	Phenol	H	0.7 ± 0.0	1.1 ± 0.2
2	2-Methylphenol	H	0.6 ± 0.1	ND
3	4-Methylphenol	H	0.6 ± 0.0	0.3 ± 0.1
4	Guaiacol	G	4.1 ± 0.2	2.1 ± 0.1
5	2,4-Dimethylphenol	H	0.1 ± 0.0	ND
6	4-Ethylphenol	H	0.6 ± 0.1	0.6 ± 0.0
7	4-Methylguaiacol	G	1.0 ± 0.1	ND
8	Benzofuran, 2,3-dihydro-	G	3.6 ± 0.1	2.5 ± 0.1
9	4-Ethylguaiacol	G	1.6 ± 0.1	ND
10	4-Methylguaiacol	G	0.6 ± 0.0	0.4 ± 0.0
11	4-Vinylguaiacol	G	4.9 ± 0.1	1.8 ± 0.1
12	4-Hydroxybenzaldehyde	H	0.7 ± 0.1	ND
13	4-(2-Propenyl)phenol	H	0.4 ± 0.0	ND
14	Syringol	S	4.4 ± 0.1	2.8 ± 0.1
15	4-Hydroxy-3-methoxybenzyl alcohol	G	0.7 ± 0.0	ND
16	2-Methoxy-5-propenyl-Phenol	G	0.2 ± 0.0	0.4 ± 0.0
17	Vanillin	G	0.5 ± 0.0	0.8 ± 0.1
18	4-Allylguaiacol	G	0.4 ± 0.0	0.2 ± 0.0
19	4-Hydroxy-3-methoxy-Benzoic acid	G	1.6 ± 0.0	1.0 ± 0.1
20	(4-Hydroxy-3-methoxyphenyl)acetone	G	0.3 ± 0.0	0.3 ± 0.0
21	1,2,3-Trimethoxy-5-methylbenzene	S	1.8 ± 0.1	ND
22	Homovanillyl alcohol	G	0.7 ± 0.0	0.2 ± 0.0
23	3,5-Dimethoxyacetophenone	S	1.9 ± 0.1	ND
24	4-Allylsyringol	S	0.3 ± 0.0	0.1 ± 0.0
25	4-Allylsyringol	S	0.3 ± 0.1	0.1 ± 0.0
26	Syringaldehyde	S	0.3 ± 0.0	0.3 ± 0.0
27	4-Allylsyringol	S	1.0 ± 0.1	0.2 ± 0.1
28	Acetosyringone	S	0.6 ± 0.0	0.5 ± 0.0
29	3,5-Dimethoxy-4-hydroxyphenylacetic acid	S	0.4 ± 0.0	0.4 ± 0.0
30	3,5-Dimethoxy-4-hydroxycinnamaldehyde	S	0.1 ± 0.0	ND
Total peak areas of H			3.6 ± 0.1	2.0 ± 0.1
Total peak areas of G			20.2 ± 1.5	9.6 ± 0.9
Total peak areas of S			11.0 ± 0.3	4.4 ± 0.2

*S* syringyl type lignin derivatives, *G* guaiacyl type lignin derivatives, *H p*-hydroxy phenylpropane

The basic analysis of lignin structure using Py–GC/MS revealed that the selective white-rot fungus *P. vitreus* possesses strong demethoxylation properties and is a powerful tool for delignification. It may provide an efficient system for exploring new and effective delignification strategies.

### Purification and characterization of extracellular VP and Lac

To verify the effect of ligninolytic enzymes in lignin depolymerization by *P. vitreus*, we purified and characterized the two ligninolytic enzymes we identified. As summarized in Additional file 2, Lac and VP produced by *P. vitreus* were successfully purified by the use of hydrophobic and ion-exchange chromatography after fractionation by ammonium sulfate precipitation. The specific activity of Lac and VP increased from 3.2 and 3.7 U/mg protein to 147.7 and 126.4 U/mg protein, respectively. The final yields of Lac and VP were 22.2 and 20.4% with a purification factor of 46.2- and 34.2-fold, respectively. After completion of the purification steps, both Lac and VP extracts showed a single band on both SDS-PAGE and native-PAGE (Additional file 3). The apparent molecular mass of Lac and VP in *P. vitreus* were estimated to be around 54.8 and 50.5 kDa, respectively, based on SDS-PAGE. For native-PAGE, the 21-day culture crude extract only showed two bands: one was Lac and the other was VP, which was consistent with assaying the enzyme activity in the crude extract. This implied that Lac and VP were the main ligninolytic enzymes present during pretreatment of corn stover by *P. vitreus*.

**Fig. 2:**
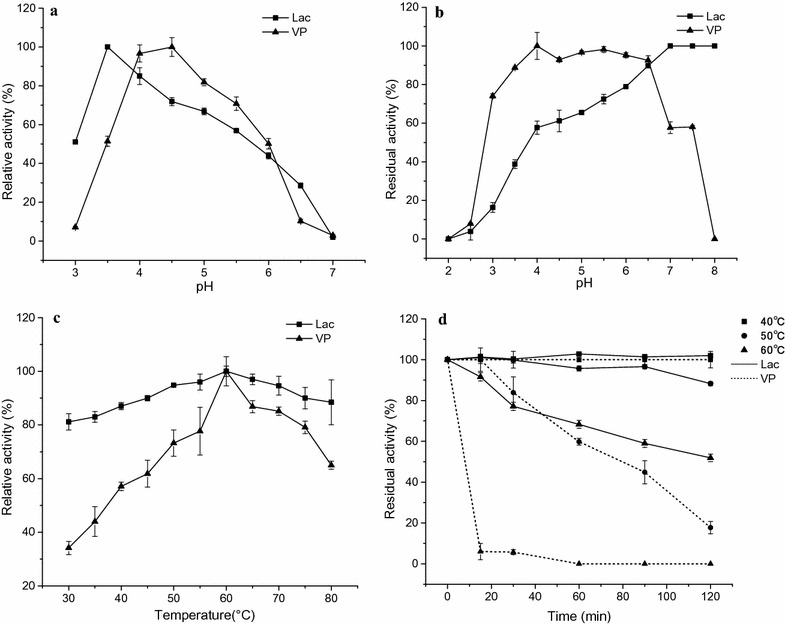
Effects of pH and temperature on the activity and stability of the purified Lac and VP. **a** Optimum pH; **b** pH stability after incubation for 24 h; **c** optimum temperature; **d** thermal stability at 40, 50, and 60 °C. The pH and temperature at which the enzyme retained the maximum residual activity was taken as 100%. Error bars shown are standard deviations of triplicate samples

The pH optima of Lac and VP were pH 3.5 and 4.5, respectively (Fig. 2a). Stability studies showed that VP was stable at a pH range of 3.0–7.5, and more than 50% of activity remained after 24 h, while Lac was stable at a pH of 4.0–8.0, and more than 50% of activity remained after 24 h (Fig. 2b). At acidic and neutral pH, VP retained much more residual activity than Lac. In addition, VP was highly stable at its optimum pH, indicating a great potential for biotechnological applications. The optimal temperature of both Lac and VP was 60 °C (Fig. 2c), which is similar to most ligninolytic enzymes [48]. Thermal stability studies showed that Lac retained 70% of its activity after incubation at 60 °C for 1 h and was stable at and below 50 °C after incubation for 2 h. VP was stable at and below 40 °C, and retained 60% of its activity after incubation at 50 °C for 1 h; however, VP activity decreased drastically at 60 °C (Fig. 2d). Thermal stability at higher temperature in enzyme’s industrial application is advantageous, and surely the type of application would demand longer incubation times than 2 h with an in vitro enzyme preparation. Therefore, we further investigated that both Lac and VP retained more than 40 and 95% enzymatic activity at 40 °C and room temperature, respectively, after incubation for 2 days.

The kinetic parameters for oxidation of substrates including 2,2′-azino-bis (3-ethylbenzothiazoline-6-sulfonic acid) (ABTS), 2,6-dimethylphenol (2,6-DMP), guaiacol, MnSO_4_, H_2_O_2_, RB5, and VA, by purified VP and Lac are included in Table 2. Lac from *P. vitreus* oxidized the typical laccase substrates ABTS, 2,6-DMP, and guaiacol, and the highest affinity was observed in ABTS, as reported for many other fungal laccases [19]. VP showed the lowest *K*_*m*_ value (14.2 μM) for RB5, indicating that it had the highest affinity towards RB5. Moreover, *P. vitreus* VP showed a higher affinity towards VA (*K*_*m*_ = 213 μM) than the VPs found in *P*. *eryngii* (*K*_*m*_ = 3000 μM) [15] and *B. adusta* (*K*_*m*_ 4000 μM) [50]. A typical lignin polymer is 10–15% phenolic in composition, while the remaining 85–90% consist of nonphenolic polymer, which possesses a high redox potential [16]. RB5 and VA represent the high redox potential compounds and nonphenolic lignin model compounds. The high reactivity of VP with high oxidation–reduction potential compounds such as RB5 and VA indicates that VP has a strong ability for delignification.

**Table 2:** Substrate specificities of Lac and VP purified from *P. vitreus*

Substrate	Wavelength (nm)	Molar extinction coefficient *ε* (L mol cm^−1^)	*K*_*m*_ (mol L^−1^)		*V*_max_ (μmol L^−1^ min^−1^)		*K*_cat_ (s^−1^)		*K*_cat_/*K*_*m*_ (s^−1^ mM^−1^)	
			Lac	VP	Lac	VP	Lac	VP	Lac	VP
ABTS	420	36,000	1.3 × 10^−5^	3.5 × 10^−5^	16.8	32.3	22.8	17.2	1791.9	487.5
2,6-DMP	470	49,600	2.8 × 10^−4^	4.4 × 10^−5^	5.2	66.7	7.1	35.5	25.1	804.2
Guaiacol	465	12,100	9.0 × 10^−4^	1.3 × 10^−4^	1.7	85.5	2.2	45.5	2.5	347.1
MnSO_4_	270	11,590	–	5.4 × 10^−5^	–	18.2	–	9.7	–	179.1
H_2_O_2_	270	11,590	–	4.4 × 10^−5^	–	39.7	–	21.1	–	475.4
RB5	598	59,800	–	1.4 × 10^−5^	–	14.5	–	7.7	–	543.2
VA	310	4700	–	2.1 × 10^−4^	–	23.1	–	12.3	–	57.7

The redox catalytic potentials of VP and Lac were determined by cyclic voltammetry and the CV data were shown in the Additional file 4. The redox potential of Lac in this study is 0.578 eV, as reported for many other fungal laccases (0.48–0.78 eV) [15]. The redox potential of VP in this study is 1.131 eV, similar to many other reported fungal VPs (>1.0 eV) [51]. Compared to Lac, VP is a high redox potential enzyme with oxidative activity on a wide variety of substrates, which contain low and high redox potential compounds, phenolic and nonphenolic lignin units [23, 24]. These properties of VP make this enzyme more suitable than Lac for delignification in biorefinery processes.

### Oxidation of lignin model compounds by purified VP and Lac from *P*. *vitreus*

**Fig. 3:**
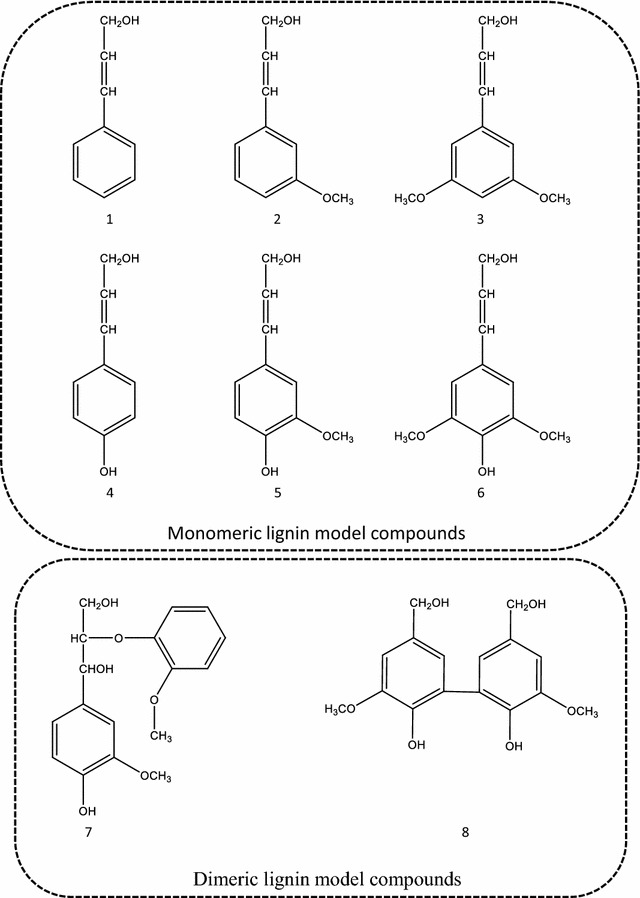
Lignin model compounds used in this study: (1) Cinnamic acid (H), (2) 3-methoxy cinnamic acid (G), (3) 3,5-dimethoxy cinnamic acid (S), (4) *p*-coumaric acid (H), (5) ferulic acid (G), (6) sinapic acid (S), (7) guaiacylglycerol β-guaiacyl ether (β-*O*-4 dimer), and (8) dehydrodivanillic alcohol (5-5′ dimer)

Six monomeric lignin model compounds and two dimeric lignin model compounds (Fig. 3) were used to study the reactions of Lac and VP with lignin: cinnamic acid (1), 3-methoxycinnamic acid (2), 3,5-dimethoxycinnamic acid (3), p-coumaric acid (4), ferulic acid (5), sinapic acid (6), guaiacylglycerol β-guaiacyl ether (7), and dehydrodivanillic alcohol (8). These monomeric and dimeric lignin model compounds represent substructures and linkages similar to those found in native lignin, respectively.

The six monomeric lignin model compounds (Fig. 31–6), which represented three types of units in the lignin structure, are referred to as H, G, and S [52]. These compounds were divided into phenolic and nonphenolic structures and differ in their methoxylation levels. Both Lac and VP could oxidize the three types of phenolic monomeric lignin model compounds (4–6) completely. In addition, VP was able to oxidize nonphenolic monomeric lignin model compounds partially, and was able to degrade 20.6 ± 1.8% of the H- (1), 27.4 ± 1.1% of the G- (2), and 39.3 ± 1.2% of the S-type compound (3) after 48 h (Table 3). It appears that the more methoxy groups the lignin model compound contained, the higher the degradation ratio was [53]. This is consistent with the observation that analysis of lignin structure by Py–GC/MS as aforementioned, which revealed that fungal pretreatment predominantly led to the degradation of S-type lignins, followed by G- and H-type ones.

**Table 3:** Monomeric lignin model compounds degradation by Lac and VP of *P. vitreus*

Phenolic lignin model compounds			Lignin structure types	Nonphenolic lignin model compounds		
Substrate^a^	Different enzyme addition	Degradation ratio (%)		Substrate^a^	Different enzyme addition	Degradation ratio (%)
*p*-coumaric acid (4)	VP	97.4 ± 1.4	H	Cinnamic acid (1)	VP	20.6 ± 1.8
	Lac	98.7 ± 2.1			Lac	0.0 ± 0.0
Ferulic acid (5)	VP	98.2 ± 1.0	G	3-Methoxy cinnamic acid (2)	VP	27.4 ± 1.1
	Lac	98.2 ± 0.9			Lac	0.0 ± 0.0
Sinapic acid (6)	VP	99.3 ± 0.2	S	3,5-Dimethoxy cinnamic acid (3)	VP	39.3 ± 1.2
	Lac	99.2 ± 1.97			Lac	0.0 ± 0.0

^a^Numbers corresponding to compounds in Fig. 3 are included in brackets

Lac had no effect on nonphenolic monomeric lignin model compounds; however, VP could oxidize both phenolic and nonphenolic lignin units. Differences in substitution were expected to cause differences in the redox potentials of the lignin model compounds, which might have been reflected in the reactivity with the two different ligninolytic enzymes [53]. Laccases have a low redox potential, and it does not have sufficient energy to extract electrons from the nonphenolic aromatic substrates [54]. VP combined the catalytic properties of both LiP and MnP with high redox potential [20], which broadens the substrate specificity of VP and enabled it to attack both phenolic and nonphenolic compounds, which comprise 80–90% of the lignin [55].

To investigate the ability of VP and Lac to break linkages in lignin, two model dimers, β-*O*-4 (7) and 5-5′ (8), were used, which represent the most abundant (more than 50% of all interunit linkages) and the most resistant (C–C linkage) interunit linkages in native lignin, respectively [13, 19]. Additional file 5a shows the HPLC chromatograms for the reaction of β-*O*-4 dimer with VP; the amount of substrate decreased dramatically upon treatment with VP, and two new major peaks were observed. Analysis of these two new peaks at 4.5 min and 5.8 min by LC–ESI–MS gave *m/z* values of 637 [M − H]^−^ and 183 [M + H]^+^, respectively (Table 4), which indicates that the oxidization of β-*O*-4 dimer by VP may occur via depolymerization to a monomer and polymerization to a tetramer simultaneously. Sale and Kenneth [56] recently reported that VP could convert β-*O*-4 lignin dimer to monomeric products, but this reaction was competing with repolymerization. By optimizing the reaction conditions, such as VP loading, H_2_O_2_ concentration, and pH, the equilibrium between depolymerization and polymerization could be controlled to a certain extent [56]. Additional file 5 b illustrates the HPLC chromatograms for the reaction progress of 5-5′ dimer with VP; the amount of 5-5′ dimer decreased dramatically upon treatment with VP and a series of new peaks was observed. Analysis of the new major peaks at 4.5, 5.2, 5.7, 6.3, and 8.4 min by LC–MS gave *m/z* values of 153 [M − H]^−^, 167 [M − H]^−^, 303 [M − H]^−^, 151 [M − H]^−^, and 301 [M + H]^+^, respectively (Table 4). This indicates that the degradation of 5-5′ dimer by VP occurs not only via the oxidation of side chains but also via cleavage of the 5-5′ linkage, which generated monomeric products, such as vanillic alcohol, vanillic aldehyde, and vanillic acid. To be representative of more condensed lignin structures, many researchers have studied 5-5′ model compounds, which resulted in bond cleavage through oxidative side-chain reactions, but no cleavage of the actual 5-5′ bond [19, 57, 58]. Lignin with relatively high numbers of C–C linkages compared to ether linkages are often referred to as condensed lignin [59]. Condensed lignin is frequently more rigid and less prone to degradation. In this study, we first found VP could cleave the actual 5-5′ linkage in vitro. In contrast, treatment of both β-*O*-4 and 5-5′ dimer with Lac led to the formation of precipitates, and then the reaction of supernatants with Lac was analyzed by HPLC which did not detect any compounds. These results indicated that Lac could not depolymerize the dimer to monomers. The formation of precipitates was due to the fact that Lac leads to polymerization of β-*O*-4 and 5-5′ dimer, which were consistent with previous reports. Ramalingam [18] treated the β-*O*-4 dimer with two types of Lac, and both treatments led to polymerization and the formation of precipitate; Lahtinen [60] also reported that polymerization of oligomers is a well-known reaction in the Lac oxidation of β-*O*-4 guaiacylic lignin model compounds. Lac caused polymerization via phenoxy radical coupling [61, 62], and the most likely structure formed by C–C coupling of the free phenolic coupling would be the biphenyl structure [18, 60, 61]. Phenolic 5-5′ dimer can also further polymerize via phenoxy radical coupling to precipitate [57, 60]. Compared with the polymerization of β-*O*-4 and 5-5′ dimer by Lac, VP could break both β-*O*-4 and 5-5′ linkages, which are the predominant linkage type and the most difficult-to-degrade linkage type in native lignin, respectively. In summary, these remarkable ability of VP to degrade lignin might be a key factor that confers *P. vitreus* its high efficiency (hydrolysis yield of cellulose was 91.5% after *P. vitreus* pretreatment) as a pretreatment system.

**Table 4:** Molecular ions detected by LC–ESI–MS for the main products of dimeric lignin model compounds in VP-catalyzed reactions

Substrate	Retention time (min)	Product peak^a^	*m/z*	Proposed products
7 (β-*O*-4 dimer)	4.5	A	637 [M − H]^−^	β-*O*-4 tetramer
	5.8	B	183 [M + H]^+^	Dihydroconiferyl alcohol
8 (5-5′ dimer)	4.5	C	153 [M − H]^−^	Vanillic alcohol
	5.2	D	167 [M − H]^−^	Vanillic acid
	5.7	E	303 [M − H]^−^	2′,6-Dihydroxy-5′-(hydroxymethyl)-3′,5-dimethoxy-[1,1′-biphenyl]-3-carbaldehyde
	6.3	F	151 [M − H]^−^	Vanillic aldehyde
	8.4	G	301 [M − H]^−^	Dehydrodivanillic aldehyde

^a^Product peaks marked on HPLC chromatograms in Fig. 4

### Enhancement of the enzymatic hydrolysis of corn stover by VP from *P*. *vitreus*

**Fig. 4:**
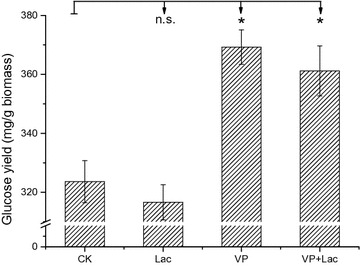
Glucose yields of corn stover during simultaneous ligninolytic enzyme treatments and hydrolysis. The following enzyme combinations were used: commercial cellulase only (CK), commercial cellulase supplemented with purified Lac from *P. vitreus* (Lac), commercial cellulase supplemented with purified VP from *P. vitreus* (VP), and commercial cellulase supplemented with both Lac and VP (VP + Lac) (*P < 0.05; *n.s*. not significant)

We next investigated the effectiveness of VP and Lac in corn stover bioconversion by including these ligninolytic enzymes in the enzymatic hydrolysis step. As shown in Fig. 4, the hydrolysis of the lignocellulosic material using VP as a supplement to the commercial cellulase significantly improved the yield of glucose by 14.1% compared to cellulase alone (*P* < 0.05), but Lac has no effect on the hydrolysis (*P* > 0.05). This is consistent with the observation that Lac and VP have different ability in degradation of lignin model compounds investigated above. The VP used in this study could oxidize nonphenolic lignin units and break main linkages in lignin, which possibly improves the accessibility of cellulase to cellulose within the biomass [2, 3, 63]. In contrast, Lac could not oxidize nonphenolic lignin units and break main linkages in lignin, which limited delignification of Lac to reduce the enzymatic recalcitrance of biomass. Moreover, when commercial cellulase was supplemented with both Lac and VP together, the yield of glucose was similar to that with VP supplementation alone (*P* > 0.05). VP improved and Lac had no effect on the enzymatic hydrolysis of corn stover, which further indicated that VP is a key enzyme in the degradation of lignin from *P. vitreus*.

In recent years, many studies have demonstrated that white-rot fungal pretreatment can enhance the enzymatic hydrolysis of lignocellulose, which are due to versatile delignification strategies of white-rot fungi [31]. Its efficiency in these processes has been mainly attributed to the release of different ligninolytic enzymes [12, 13]. However, it remains unclear which fungal delignification strategy based on ligninolytic enzyme plays a key role in unlocking the recalcitrant structure of lignocellulase and improves the enzymatic hydrolysis greatly. A detailed understanding of fungal delignification strategy will contribute to the development of lignocellulose biorefinery strategies based on the ligninolytic enzymes from white-rot fungi. Fungal pretreatment has some advantages, it is low-cost, environmentally friendly, and does not produce inhibitors to fermentation, while the potential loss in sugar content and long incubation times hinder its industrial applications [64, 65]. Therefore, the treatment with the effective ligninolytic enzymes from fungi in vitro was imperative. Bio-treatment with the VP in vitro enhances the glucose yield after hydrolysis similar to fungal pretreatment but without the consumption of sugar during the pretreatment process. We are the first to report that VP can improve the enzymatic hydrolysis of corn stover in vitro. This opens the way to seek more efficient VPs for the enhancement of enzymatic hydrolysis.

## Conclusions

In the study, firstly, a new fungal isolate, *Physisporinus vitreus* was found to show a strong potential to enhance the enzymatic hydrolysis of lignocellulosic biomass. After pretreatment with *P. vitreus*, the saccharification of corn stover was significantly enhanced to 349.1 mg g^−1^ biomass (yield of glucose) and 91.5% (hydrolysis yield of cellulose), as high as levels reached by thermochemical treatment [28, 29] and reaches levels similar to that of biomass pretreated using thermochemical processes [5, 6, 30]. Analysis of the lignin structure after pretreatment with *P. vitreus* using Py–GC/MS, revealed significant demethoxylation and lignin degradation by *P. vitreus*, suggesting that *P. vitreus* is an efficient lignin-degrading fungus. Then study of extracellular ligninases from this highly efficient pretreatment system, we found a novel delignification strategy was based on VP, which could oxidize both phenolic and nonphenolic lignin units and break different linkages in lignin. Moreover, VP improved the enzymatic hydrolysis of corn stover in vitro reached to the level of biological treatment. On this basis, a new and efficient VP treatment system was established, and the role of VP in biological pretreatment also was verified. Moreover, we firstly indicated that VP can improve the enzymatic hydrolysis of corn stover in vitro.

Overall, the VP from *P. vitreus* in this study shows a potential use in lignin depolymerization, which makes it a really interesting enzyme for biotechnological applications, such as production of biofuel and other high value-added bio-based materials.

## Methods

### Fungus and inoculum preparation

*Physisporinus vitreus* was isolated from stone seam at the Huazhong University of Science and Technology and identified based on its macro- and microscopic morphology. The correct identity of *P. vitreus* was further confirmed by comparing the nucleotide sequence of the internal transcribed spacer (ITS) region of the nuclear ribosomal RNA gene with that of related strains in the GenBank database [66, 67]. The GenBank Accession Number of *P. vitreus* is KU958554. *P. vitreus* was cultured on potato extract agar slants for 7 days at 28 °C. Five disks (1 cm in diameter) of inocula were grown on potato extract broth for 5 days (150 rpm, 28 °C). This new isolate belongs to *Physisporinus* sp. which has no potential hazards for plants and has been reported for engineering value-added wood products [27]. The potential hazards of this isolate for animals and the human have not been reported.

### Pretreatment

Corn stover was collected from HuBei (China) and dried at 40 °C in an oven for 3 days. Raw corn stover was smashed to pass through a 0.45-mm screen. The biological pretreatments with *P. vitreus* were carried out in 250-mL Erlenmeyer flasks with 2 g ground corn stover and 5 mL distilled water with 0.01 mmol L^−1^ MnSO_4_. Flasks were sterilized in the autoclave for 40 min at 121 °C and aseptically inoculated with 2-mL fungal inocula. Cultures were maintained statically at 28 °C for 1, 2, 3, 4, and 5 weeks and then dried at 60 °C for 3 days for the following saccharification and chemical component analysis.

### Enzymatic hydrolysis of corn stover

Enzymatic hydrolysis of corn stover (raw and fungal-treated) were performed at 2% substrate concentration (w/v) in 50 mmol L^−1^ sodium acetate buffer (pH4.8). Cellulase (C9748, Sigma-Aldrich) was added at an enzyme loading of 30 FPU (filter paper unit)/g dry substrate. To prevent microbial contamination, sodium azide (0.05%, w/v) was added to the mixture. The samples were incubated in a controlled reciprocal shaking water bath at 50 °C, 150 rpm for 72 h. The glucose concentration was measured by high-performance liquid chromatography (HPLC) system (model 1000, Shimadzu, Japan) using a column (Sugar Pak 1; Waters Ltd, Milford, US) and a refractive index (RI) detector. The mobile phase used was deionized water. The column was used at a flow rate of 0.6 mL/min and a column temperature of 75 °C. The glucose yields were calculated using the following equations:

1 $$\text{Glucose yield (mg}\,\text{g}^{ - 1} \text{)} = \tfrac{{\text{mg of glucose after enzymatic hydrolysis}}}{{\text{g of raw corn stover}}}$$

2 $${{\text{Hydrolysis yield of cellulose (\% )}}} = \tfrac{{\text{g of glucose after enzymatic hydrolysis} \;\times \;\text{100}}}{{\text{g of glucan }{\text{in}}\text{ raw corn stover}\; \times \;1.11}}.$$

Sample weight loss was taken into account for yield estimation; all results were calculated from the original biomass.

### Chemical component analysis of corn stover

The contents of acid soluble lignin (ASL), acid insoluble lignin (AIL), cellulose, and hemicellulose in all samples were determined based on the “determination of structural polysaccharides and lignin in biomass (Version 2006)” from the National Renewable Energy Laboratory (NREL). Lignin content was the summation of ASL and AIL contents [68].

### Enzyme extraction and assay

To obtain enzyme extracts, sodium acetate buffer solution (50 mmol L^−1^, pH 4.8; 1:50 w/v) was added to solid cultures of *P*. *vitreus* after different pretreatment periods. The flasks were then shaken at 150 rpm for 1 h. The contents were filtered and centrifuged at 10,000×*g* for 15 min to get a clear supernatant, which were the extracellular enzyme extracts.

Lac, LiP, and MnP were analyzed in the enzyme extract as previously described [48]. In addition, VP activity was assayed by monitoring the oxidation of 1 μmol RB5 (Sigma-Aldrich) in 100 mmol L^−1^ sodium tartrate at pH 3 (*ε*_598_ = 47,600 L mol^−1^ cm^−1^) in the presence of 0.1 mmol L^−1^ H_2_O_2_. The total protein concentration of each sample was measured by the BCA Protein Assay Kit (Beyotime Institute of Biotechnology, China) following the manufacturer’s instructions.

### Py–GC/MS analysis

Analytical pyrolysis of corn stover was performed using a Pyroprobe 5200 analytical pyrolyzer (CDS Analytical Inc.) at 500 °C for one minute. The volatile pyrolysis products were analyzed using a gas chromatograph (Agilent 7890A) equipped with a mass spectrometer (5975CMSD) and HP-5MS column using the following chromatograph: 3 min isothermal at 40 °C, followed by 5 °C min^−1^ to 150 °C, 10 °C min^−1^ to 250 °C, and a final isothermal step at 250 °C for 25 min. The MS was performed under 70 eV EI conditions with a range from 29 to 600 *m/z*. The pyrolysis products were identified based on the reported literature and by comparing the mass spectra with the NIST mass spectrum library [69, 70]. Peak molar areas were calculated for the corn stover-derived products, the summed areas were normalized to 100, and the data for three repetitive analyses were averaged and expressed as percentages.

### Purification and characterization of extracellular Lac and VP

At the peak of Lac and VP activity, crude enzyme extracts were harvested as described previously. The cell-free filtrates were then subjected to ammonium sulfate fractionation. First, crude Lac and VP extracts were brought to 40 and 30% saturation, respectively, by gradual addition of ammonium sulfate. Then, the precipitated proteins were removed by centrifugation at 10,000×*g* for 15 min, and more solid ammonium sulfate was added to achieve 80 and 70% saturation for crude Lac and VP extracts, respectively, followed by keeping for overnight at 4 °C. On the next day, the precipitates were collected by centrifugation at 10,000×*g* for 15 min and dissolved in 20 mmol L^−1^ sodium acetate buffer (pH 4.8). After dialysis and concentration, these crude Lac and VP solutions were purified by alternate use of ion-exchange and hydrophobic chromatography equilibrated with 20 mmol L^−1^ sodium acetate buffer at pH 4.8 [48]. The fractions with Lac and VP activity were collected, concentrated, and dialyzed by ultrafiltration. The purity of Lac and MnP was confirmed by SDS-PAGE and native-PAGE, which were performed on a 5% stacking gel and a 12% resolving gel in a mini-electrophoresis apparatus (BioRad, USA). The protein bands were visualized by staining with Coomassie Brilliant Blue R-250 in SDS-PAGE and staining with ABTS and H_2_O_2_ in native-PAGE. The molecular weights of purified Lac and VP were estimated by SDS-PAGE in comparison to prestained standard proteins (15–170 kDa). Moreover, we conducted the following studies using these purified Lac and VP.

To study the optimum pH, the Lac activity on ABTS, and the VP activity on MnSO_4_ were individually determined in the 2 malonate buffer at a pH ranging from 3.0 to 7.0 at 25 °C. The pH stability was investigated in potassium chloride/hydrochloric acid buffer (pH range from 1.0 to 2.0) and citrate–phosphate buffer (pH range from 2.5 to 8.0) by incubating the enzymes for 24 h at 30 °C, followed by activity measurements. The optimal temperature for Lac and VP activity was determined from 30 to 80 °C. The thermal stability was measured by incubating Lac and VP at a temperature range of 40–60 °C for 2 h with samples taken periodically for activity measurements. All measurements were made in triplicate.

To determine the substrate specificities of Lac and VP, the oxidation of different substrates, ABTS, DMP, guaiacol, MnSO_4_, H_2_O_2_, RB5, and VA was studied in 50 mmol L^−1^ malonate (pH 4.5) and 0.1 mmol L^−1^ H_2_O_2_ with or without 1 mmol L^−1^ MnSO_4_. The activities of Lac and VP were determined spectrometrically using the corresponding wavelengths and absorption coefficients for each substrate [71, 72] (Table 3).

The oxidation–reduction potentials of Lac and VP were determined by Cyclic voltammetry (CV) experiments [73]. CV experiments were performed with an electrochemical workstation (CHI600E, Shanghai Chenhua Instruments Limited, China) attached to an analytical three-electrode configuration. All measurements were carried out in a 25-mL cell at room temperature. A glassy carbon electrode (GCE) with deposited enzyme film (Lac or VP) was used as a working electrode. A platinum mesh (2 cm × 2 cm) was used as the counter-electrode and a saturated calomel electrode (SCE) served as a reference electrode. Before each experiment, the surface of the glassy carbon electrode was polished on a diamond-polishing pad followed by washing with distilled water. To prepare a working electrode, a 200 μL of enzyme solution in acetate buffer solution (0.1 mg/mL) was dropped onto the polished surface of the GC working electrode and allowed to dry for 15 min at room temperature. All CVs were recorded in 0.1 M acetate buffer solution (pH = 4.8) at 25 °C. The electrode potential was scanned from −0.9 to 0.4 V at scan rate of 50 mV/s for Lac and scanned from −1.5 to 0.4 V at scan rate of 10 mV/s for VP for three cycles, respectively. Glassy carbon electrode (GCE) in aqueous solutions is considered to be an inert electrode for hydronium ion reduction.

### Oxidation of lignin model compounds by purified Lac and VP from *P*. *vitreus*

The substrates used are summarized in Fig. 3 and included six monomeric (purchased from Sigma-Aldrich) and two dimeric lignin model compounds (synthesis according to Additional files 6, 7). Each lignin model compound was added at a final concentration of 1 mmol L^−1^ to the reaction mixtures. For VP, the reaction mixtures consisted of VP (1 U mL^−1^), H_2_O_2_ (0.1 mmol L^−1^), and MnSO_4_ (1 mmol L^−1^) in 50 mmol L^−1^ malonate buffer at pH 4.5, and additional H_2_O_2_ was supplemented every 8 h. The Lac reaction mixtures consisted of Lac (1 U mL^−1^) in 50 mmol L^−1^ sodium acetate buffer at pH 4.8. The reaction mixtures were incubated with the different lignin substrates at 30 °C for 48 h, and the residual concentrations of the lignin model compounds were analyzed by HPLC using a reversed phase C_18_-column (WondaSil-C_18_, 4.6 mm × 250 mm, 5 μm). Methanol–water (65:35, v/v) containing 0.1% formic acid was used as an eluate, and compounds were detected by UV at 270 nm. For each test, 10 μL of sample was injected and eluted at a flow rate of 1 mL min^−1^. The oxidation products of lignin dimers treated with VP were further analyzed via LC–ESI–MS. Chromatographic separation was performed using the same column as above at 40 °C with a flow rate of 0.8 mL min^−1^ and an injection volume of 30 μL. The eluate was methanol–water (55:45, v/v) containing 0.1% formic acid, and reaction products were detected by a UV detector at 270 nm. LC–ESI–MS analyses were conducted in positive and negative ionization modes simultaneously. The drying gas was operated at a flow rate of 10 mL min^−1^ at 280 °C. The nebulizer pressure and the capillary voltage were 40 psig and 3500 V, respectively.

### Enzymatic hydrolysis of corn stover supplementation with Lac and VP from *P*. *vitreus*

In order to make the enzyme and raw materials in full contact and maximize the effect, the unpretreated corn stover used here was further ground, which may lead to higher glucose yield of control. The previous raw corn stover was ground in a 500-mL ceramic jar with 10 × 10-mm ceramic ball bearings using a planetary ball mill. The total ball-milling time was 2 h at 400 rpm with 5-min breaks after every 10 min of milling. To test the effectiveness of different enzyme mixtures in corn stover hydrolysis, 5% (w/v) corn stover was added to 50 mmol L^−1^ sodium citrate buffer (pH 4.8) and incubated at 30 °C at 180 rpm for 48 h with the following enzyme mixtures: (i) commercial cellulase (30 FPU/g substrate) (Sigma-Aldrich); (ii) commercial cellulase plus purified Lac (100 U/g substrate); (iii) commercial cellulase plus purified VP (100 U/g substrate); and (iv) commercial cellulase plus purified Lac and VP (100 U/g substrate each); glucose oxidase (0.1 U/mL) was added to all samples to supply H_2_O_2_. The release of fermentable sugar was quantified as described previously [28].

### Statistical analysis

All samples were tested in triplicate. Mean values are presented with their standard errors. Multiple comparison tests were performed with ANOVA (∗*P* < 0.05; n.s., not significant). The experimental results were validated by statistical analysis using Origin 8.0 software.

## Additional files

**Additional file 1.** Py–GC/MS chromatograms of (a) untreated corn stover and (b) corn stover pretreated with *P. vitreus* for 28 day.

**Additional file 2.** Summary of the purification of Lac and VP from *P. vitreus.*

**Additional file 3.** SDS-PAGE (a) and Native-PAGE (b) of purified VP and Lac from *P. vitreus.*

**Additional file 4.** Cyclic voltammograms of Lac and VP.

**Additional file 5.** HPLC chromatograms of products of dimeric lignin model compounds 7 (a) and 8 (b) in VP-catalyzed reactions.

**Additional file 6.** Synthesis scheme of guaiacylglycerol β-guaiacyl ether.

**Additional file 7.** Synthesis scheme of dehydrodivanillyl alcohol.

## Abbreviations

*P. vitreus*: *Physisporinus vitreus*
VP: versatile peroxidase
Lac: laccase
LiP: lignin peroxidase
MnP: manganese peroxidase
ABTS: 2,2′-azino-bis (3-ethylbenzothiazoline-6-sulfonic acid)
DMP: 2,6-dimethylphenol
VA: veratryl alcohol
RB5: reactive black 5
H: *p*-hydroxyphenyl
G: guaiacyl
S: syringyl
Py–GC/MS: Pyrolysis–gas chromatography/mass spectrometry
HPLC: high-performance liquid chromatography
LC–ESI–MS: high-performance liquid chromatography–electrospray ionization–mass spectrometry
