# Supplementary material for: A novel and efficient fungal delignification strategy based on versatile peroxidase for lignocellulose bioconversion
Source: Biotechnol Biofuels. 2017 Sep 13;10:218. doi: 10.1186/s13068-017-0906-x (PMC5598073; doi:10.1186/s13068-017-0906-x)
Supplement: Supplementary file 1 — Additional file 1. Py–GC/MS chromatograms of (a) untreated corn stover and (b) corn stover pretreated with P. vitreus for 28 day. [file 13068_2017_906_MOESM1_ESM.docx]

**Additional file 1:** Py-GC/MS chromatograms of (a) untreated corn stover and (b) corn stover pretreated with *P. vitreus* for 28 d
