# Supplementary material for: A novel and efficient fungal delignification strategy based on versatile peroxidase for lignocellulose bioconversion
Source: Biotechnol Biofuels. 2017 Sep 13;10:218. doi: 10.1186/s13068-017-0906-x (PMC5598073; doi:10.1186/s13068-017-0906-x)
Supplement: Supplementary file 2 — Additional file 2. Summary of the purification of Lac and VP from P. vitreus. [file 13068_2017_906_MOESM2_ESM.docx]

| Purification step | Total activity（U） | | Total protein（mg） | | Specific activity（U/mg） | | Purification (fold) | | Yield (%) | |
| --- | --- | --- | --- | --- | --- | --- | --- | --- | --- | --- |
|  | VP | Lac | VP | Lac | VP | Lac | VP | Lac | VP | Lac |
| Culture filtrate | 929.2 | 731.6 | 252.5 | 225.6 | 3.7 | 3.2 | 1.0 | 1.0 | 100.0% | 100.0% |
| (NH_4_)_2_SO_4_ precipitation | 751.9 | 589.2 | 63.9 | 59.4 | 11.8 | 9.9 | 3.2 | 3.1 | 80.9% | 80.5% |
| Hydrophobic interaction | 391.3 | 231.1 | 7.9 | 6.5 | 49.5 | 35.6 | 13.4 | 11.1 | 42.1% | 31.6% |
| DEAE ion-exchange | 189.6 | 162.5 | 1.5 | 1.1 | 126.4 | 147.7 | 34.2 | 46.2 | 20.4% | 22.2% |

**Additional file 2:** Summary of the purification of Lac and VP from *P.* *vitreus*
