# Supplementary material for: A novel and efficient fungal delignification strategy based on versatile peroxidase for lignocellulose bioconversion
Source: Biotechnol Biofuels. 2017 Sep 13;10:218. doi: 10.1186/s13068-017-0906-x (PMC5598073; doi:10.1186/s13068-017-0906-x)
Supplement: Supplementary file 3 — Additional file 3. SDS-PAGE (a) and Native-PAGE (b) of purified VP and Lac from P. vitreus. [file 13068_2017_906_MOESM3_ESM.docx]

**Additional file 3:** SDS-PAGE (a) and Native-PAGE (b) of purified VP and Lac from *P. vitreus*.
