# Supplementary material for: A novel and efficient fungal delignification strategy based on versatile peroxidase for lignocellulose bioconversion
Source: Biotechnol Biofuels. 2017 Sep 13;10:218. doi: 10.1186/s13068-017-0906-x (PMC5598073; doi:10.1186/s13068-017-0906-x)
Supplement: Supplementary file 6 — Additional file 6. Synthesis scheme of guaiacylglycerol β-guaiacyl ether. [file 13068_2017_906_MOESM6_ESM.docx]

**Additional file 6:** Synthesis scheme of guaiacylglycerol β-guaiacyl ether

**Scheme S1:** Synthesis of the phenolic β-O-4 model substrate adapted from Kawai et al. [[1](#_ENREF_1)]

**(1) 4'-Benzyloxy-3',5'-dimethoxyacetophenone (Ⅱ)**

A solution of 3',5'-dimethoxy-4'-hydroxyacetophenone（Ⅰ）(1.0g) (acetosyringone 99%; sigma) in pyridine (5ml) was cooled at 0°C with stirring; benzoyl chloride (800ul) was added under nitrogen atmosphere (white precipitate was formed). The resulting solution was stirred at 0°C for 20min. The reaction mixture was partitioned between ethyl acetate (100ml) and 1N HC1 (100ml). The organic layer was washed successively with 1N HC1 (60ml, three), 1 N NaOH (60ml, three), and saturated brine (60ml, three). It was dried over anhydrous MgSO_4_ and evaporated under reduced pressure to yield a crude 4'-benzoyloxy-3',5'-dimethoxyacetophenone (Ⅱ) (2.50g).

**(2) 1-(4-Benzoyloxy-3,5-dimethoxyphenyl)-2-bromoethanone (Ⅲ)**

To a solution of crude compound Ⅱ (2.5g) in ethyl acetate (10ml), 4-dimethylaminopyridiniumbromide perbromide (3.7g, 10.0mmol) (Tokyo Chemical Industry) was added under nitrogen atmosphere. The resulting solution was stirred at ambient temperature for 40min. The reaction mixture was partitioned between ethyl acetate (150ml) and water (100ml). The organic layer was washed successively with water (60ml, three) and saturated brine (100ml, three). It was dried over anhydrous MgSO_4_ and evaporated under reduced pressure. The residue was recrystallized from a mixture of acetone and n-hexane to give white crystals of 1- (4-benzoyloxy-3,5-dimethoxyphenyl)-2-bromoethanone (Ⅲ) (1.82g, yield 79.08% from acetosyringone), mp 163 °- 166°C. [Note: Compound Ⅲ and its analogue were also purified by column chromatography with ethyl acetate-nhexane,1:3.]

**(3) 1-(4-Benzoyloxy-3,5-dimethoxyphenyl)-2-(2,6-dimethoxyphenoxy) ethanone (Ⅳ)**

To a solution of compound Ⅲ(1.82g) in toluene (10ml), 2,6-dimethoxyphenol (1.17g), 18-crown-6-ether (1.92g), and KOH (479.1mg) was added under nitrogen atmosphere. The resulting solution was stirred at ambient temperature for 50 min.The reaction mixture was partitioned between ethyl acetate (150ml) and water (100ml). The organic layer was washed with saturated brine (100ml, three), dried over anhydrous MgSO_4_, and evaporated under reduced pressure. The residue was purified by recrystallization from ethyl acetate and n-hexane to give 1-(4-benzoyloxy-3,5-dimethoxyphenyl)-2-(2,6-dimethoxyphenoxy) ethanone(Ⅳ) (2.02g, yield 92.9%) , mp 135°-137°C.

**(4)1-(4-Benzoyloxy-3,5-dimethoxyphenyl)-2-(2,6-dimethoxyphenoxy)-3-hydroxypropane (Ⅴ)**

To a solution of compound Ⅳ(1.35g) in dimethylsulfoxide (5.0ml), paraformaldehyde (113.6 mg, 95%) and K_2_CO_3_ (124.4mg) were added under nitrogen atmosphere. The resulting solution was stirred at ambient temperature for 90 min. The reaction mixture was partitioned between ethyl acetate (100ml) and water (60ml). The organic layer was washed with saturated brine (60ml), dried over anhydrous MgSO_4_, and evaporated under reduced pressure. The residue was purified by column chromatography (eluent was ethyl acetate-n-hexane, 1:1) to give 1-(4-benzoyloxy-3,5- dimethoxyphenyl)-2- (2,6-dimethoxyphenoxy)-3-hydroxypropane (Ⅴ) (1.205g, yield 83.4%).

**(5)1-(4-Benzoyloxy-3,5-dimethoxyphenyl)-i,3-dihydroxy-2-(2,6-dimethoxyphenoxy) propane (Ⅵ)**

To a solution of compound Ⅴ (1.205g) in methanol (3ml) and tetrahydrofuran (4ml), sodium borohydride (200mg) was added at 0°C. The resulting solution was stirred at 0°C for 10min. The reaction mixture was partitioned between ethyl acetate (100ml) and water (60ml). The organic layer was washed with saturated brine (60ml, three), dried over anhydrous MgSO_4_, and evaporated under reduced pressure to give a crude mixture of 1-(4-benzoyloxy-3,5-dimethoxyphenyl)-l,3-dihydroxy-2- (2-methoxyphenoxy) propane (**Ⅵ**) (1.36g).

**(6)1,3-Dihydroxy-1-(3,5-dimethoxy-4-hydroxyphenyl)-2-(2,6-dimethoxyphenoxy) propane (Ⅶ)**

To a solution of crude compound **Ⅵ** (1.36g) in benzene (5ml), n-butylamine (2.5ml) was added under nitrogen atmosphere. The resulting solution was stirred at ambient temperature for 96h. The reaction mixture was partitioned between ethyl acetate (100ml) and 1N HC1 (60ml). The organic layer was washed with saturated brine (60ml, three), dried over anhydrous MgSO_4_, and evaporated under reduced pressure. The residue was purified by column chromatography (eluent was ethyl acetaten-hexane, 3:1) to give 1,3-dihydroxy-l-(3,5-dimethoxy-4-hydroxyphenyl)- 2-(2,6-dimethoxyphenoxy)propane (1) (986.0mg)
