# Supplementary material for: A novel and efficient fungal delignification strategy based on versatile peroxidase for lignocellulose bioconversion
Source: Biotechnol Biofuels. 2017 Sep 13;10:218. doi: 10.1186/s13068-017-0906-x (PMC5598073; doi:10.1186/s13068-017-0906-x)
Supplement: Supplementary file 7 — Additional file 7. Synthesis scheme of dehydrodivanillyl alcohol. [file 13068_2017_906_MOESM7_ESM.docx]

**Additional file 7:** Synthesis scheme of dehydrodivanillyl alcohol

**Scheme S2:** Synthesis of the phenolic 5-5’ model substrate adapted from [JC Pew](http://xueshu.baidu.com/s?wd=author%3A%28John%20C.%20Pew%29%20&tn=SE_baiduxueshu_c1gjeupa&ie=utf-8&sc_f_para=sc_hilight%3Dperson) [[2](#_ENREF_2)]

(1) 2.0 grams of vanillin dissolved in 40ml absolute ethanol and 360ml water containing 6.0mg of peroxidase, when the color of the solution changed from colorless to pale yellow, 10ml of 3.0% hydrogen peroxide was added dropwise over a period of 1 hour, the reaction system soon had brown precipitation. The mixture was allowed to stand over 12 hours at room temperature, the precipitate was filtered, washed with water, ethanol and then washed with acetone which removed the residues of vanillin. The dehydrogenation of vanillin was received over vacuum drying vacuum, the product, 0.5632g, melting point was at 306-308℃, and the yield was 28.35%.

(2) 1.0g dehydrogenation of vanillin was dissolved in 40 ml 0.5 mol/L NaOH solution, and then 0.4g NaBH_4_ was added. The reaction was for 12h at room temperature, the reaction mixture was treated with acetic acid to decompose excess NaBH_4_,the pH of solution was adjusted to 3.0 by 1M HCl. The product was isolated by centrifugation, which was washed with water until the pH was 7.0, and HBO_3_ was removed. Dehydrogenation of acetovanillone was obtained by vacuum drying, the product, 0.6256g, melting point was at 157-161℃, and the yield was 62.15%.

**Supporting References:**

1. Kawai S, Okita K, Sugishita K, Ai T, Ohash H: Simple method for synthesizing phenolic β-O -4 dilignols. *Journal of Wood Science* 1999, 45:440-443.

2. Pew JC: Evidence of a Biphenyl Group in Lignin1. *Jorgchem* 1963:1048-1054.
